# Absence of long-term structural and functional cardiac abnormalities on multimodality imaging in a multi-ethnic group of COVID-19 survivors from the early stage of the pandemic

**DOI:** 10.1093/ehjimp/qyad034

**Published:** 2023-10-26

**Authors:** Lorenzo R Sewanan, Marco R Di Tullio, Andrew F Laine, Belinda D’Souza, Jay Leb, Alexander Mironov, Ahsan Khan, Dylan E Stanger, Elisa E Konofagou, Rochelle L Goldsmith, Sachin R Jambawalikar, Cole B Hirschfeld, Michelle Castillo, Kathleen J Durkin, Stephen Dashnaw, J Thomas Vaughan, Andrew J Einstein

**Affiliations:** Department of Medicine, Columbia University Irving Medical Center/NewYork-Presbyterian Hospital, 622 West 168th Street, PH 10-203E, New York 10032, NY, USA; Department of Medicine, Columbia University Irving Medical Center/NewYork-Presbyterian Hospital, 622 West 168th Street, PH 10-203E, New York 10032, NY, USA; Seymour, Paul, and Gloria Milstein Division of Cardiology, Columbia University Irving Medical Center/NewYork-Presbyterian Hospital, 622 West 168th Street, PH 10-203E, New York 10032, NY, USA; Department of Biomedical Engineering, Columbia University Fu Foundation School of Engineering and Applied Science, New York, NY, USA; Department of Radiology, Columbia University Irving Medical Center/NewYork-Presbyterian Hospital, 622 West 168th Street, PH 10-203E, New York 10032, NY, USA; Department of Radiology, Columbia University Irving Medical Center/NewYork-Presbyterian Hospital, 622 West 168th Street, PH 10-203E, New York 10032, NY, USA; Department of Radiology, Columbia University Irving Medical Center/NewYork-Presbyterian Hospital, 622 West 168th Street, PH 10-203E, New York 10032, NY, USA; Department of Medicine, Columbia University Irving Medical Center/NewYork-Presbyterian Hospital, 622 West 168th Street, PH 10-203E, New York 10032, NY, USA; Seymour, Paul, and Gloria Milstein Division of Cardiology, Columbia University Irving Medical Center/NewYork-Presbyterian Hospital, 622 West 168th Street, PH 10-203E, New York 10032, NY, USA; Department of Medicine, Columbia University Irving Medical Center/NewYork-Presbyterian Hospital, 622 West 168th Street, PH 10-203E, New York 10032, NY, USA; Seymour, Paul, and Gloria Milstein Division of Cardiology, Columbia University Irving Medical Center/NewYork-Presbyterian Hospital, 622 West 168th Street, PH 10-203E, New York 10032, NY, USA; Department of Medicine, Columbia University Irving Medical Center/NewYork-Presbyterian Hospital, 622 West 168th Street, PH 10-203E, New York 10032, NY, USA; Seymour, Paul, and Gloria Milstein Division of Cardiology, Columbia University Irving Medical Center/NewYork-Presbyterian Hospital, 622 West 168th Street, PH 10-203E, New York 10032, NY, USA; Department of Biomedical Engineering, Columbia University Fu Foundation School of Engineering and Applied Science, New York, NY, USA; Department of Radiology, Columbia University Irving Medical Center/NewYork-Presbyterian Hospital, 622 West 168th Street, PH 10-203E, New York 10032, NY, USA; Department of Medicine, Columbia University Irving Medical Center/NewYork-Presbyterian Hospital, 622 West 168th Street, PH 10-203E, New York 10032, NY, USA; Seymour, Paul, and Gloria Milstein Division of Cardiology, Columbia University Irving Medical Center/NewYork-Presbyterian Hospital, 622 West 168th Street, PH 10-203E, New York 10032, NY, USA; Department of Radiology, Columbia University Irving Medical Center/NewYork-Presbyterian Hospital, 622 West 168th Street, PH 10-203E, New York 10032, NY, USA; Maurice R. and Corinne P. Greenberg Division of Cardiology, Weill Cornell Medicine/NewYork-Presbyterian Hospital, New York, NY, USA; Department of Medicine, Columbia University Irving Medical Center/NewYork-Presbyterian Hospital, 622 West 168th Street, PH 10-203E, New York 10032, NY, USA; Seymour, Paul, and Gloria Milstein Division of Cardiology, Columbia University Irving Medical Center/NewYork-Presbyterian Hospital, 622 West 168th Street, PH 10-203E, New York 10032, NY, USA; Department of Biomedical Engineering, Columbia University Fu Foundation School of Engineering and Applied Science, New York, NY, USA; Department of Radiology, Columbia University Irving Medical Center/NewYork-Presbyterian Hospital, 622 West 168th Street, PH 10-203E, New York 10032, NY, USA; Department of Biomedical Engineering, Columbia University Fu Foundation School of Engineering and Applied Science, New York, NY, USA; Department of Radiology, Columbia University Irving Medical Center/NewYork-Presbyterian Hospital, 622 West 168th Street, PH 10-203E, New York 10032, NY, USA; Department of Medicine, Columbia University Irving Medical Center/NewYork-Presbyterian Hospital, 622 West 168th Street, PH 10-203E, New York 10032, NY, USA; Seymour, Paul, and Gloria Milstein Division of Cardiology, Columbia University Irving Medical Center/NewYork-Presbyterian Hospital, 622 West 168th Street, PH 10-203E, New York 10032, NY, USA; Department of Radiology, Columbia University Irving Medical Center/NewYork-Presbyterian Hospital, 622 West 168th Street, PH 10-203E, New York 10032, NY, USA

**Keywords:** transthoracic echocardiography, cardiac magnetic resonance, COVID-19, cardiac inflammation, multimodality imaging

## Abstract

**Aims:**

Many patients with coronavirus disease-2019 (COVID-19), particularly from the pandemic’s early phase, have been reported to have evidence of cardiac injury such as cardiac symptoms, troponinaemia, or imaging or ECG abnormalities during their acute course. Cardiac magnetic resonance (CMR) and transthoracic echocardiography (TTE) have been widely used to assess cardiac function and structure and characterize myocardial tissue during COVID-19 with report of numerous abnormalities. Overall, findings have varied, and long-term impact of COVID-19 on the heart needs further elucidation.

**Methods and results:**

We performed TTE and 3 T CMR in survivors of the initial stage of the pandemic without pre-existing cardiac disease and matched controls at long-term follow-up a median of 308 days after initial infection. Study population consisted of 40 COVID-19 survivors (50% female, 28% Black, and 48% Hispanic) and 12 controls of similar age, sex, and race-ethnicity distribution; 35% had been hospitalized with 28% intubated. We found no difference in echocardiographic characteristics including measures of left and right ventricular structure and systolic function, valvular abnormalities, or diastolic function. Using CMR, we also found no differences in measures of left and right ventricular structure and function and additionally found no significant differences in parameters of tissue structure including T1, T2, extracellular volume mapping, and late gadolinium enhancement. With analysis stratified by patient hospitalization status as an indicator of COVID-19 severity, no differences were uncovered.

**Conclusion:**

Multimodal imaging of a diverse cohort of COVID-19 survivors indicated no long-lasting damage or inflammation of the myocardium.

## Graphical Abstract

**Graphical abstract:**
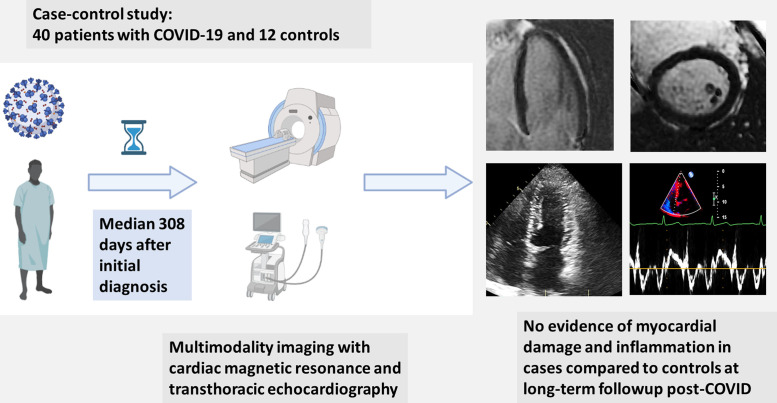

## Introduction

The ongoing global coronavirus disease-2019 (COVID-19) pandemic causes a spectrum of disease, from asymptomatic and mild infections to severe multisystem failure. The SARS-CoV-2 respiratory virus is able to cause severe inflammatory phenomena that impact many organ systems beyond the lungs, including neurologic, cardiovascular, and renal manifestations. Many patients have been reported to present with or develop markers of cardiac injury such as troponinaemia, electrocardiographic changes, cardiovascular symptoms, and echocardiographic abnormalities during their acute course of disease.^1,2^ Such acute cardiac disease manifestations in the setting of COVID-19 range from myocarditis, pericarditis, Takutsubo cardiomyopathy, ischaemia, and arrhythmia in the setting of systemic inflammation.^1–12^ Transthoracic echocardiography (TTE) has been widely used to assess cardiac function and structure during COVID-19 due to ease of access during acute illness and follow-up, with numerous reports of abnormalities. Cardiac magnetic resonance (CMR) has been particularly useful in complementing TTE for further assessment of tissue structure.^13–15^ Overall, findings are varied,^1,2,5–8,13–42^ and long-term impact of COVID-19 on myocardial structure and function needs further elucidation to characterize whether the myocardial inflammation is truly transient or could act as a substrate for development of long-term cardiac dysfunction and adverse remodelling. In our study, we performed transthoracic echocardiography (TTE) and CMR in COVID-19 patients and controls from the early phase of the pandemic at a long-term follow-up of at least 6 months post-infection, using an array of CMR techniques to comprehensively evaluate the myocardium, so as to fully appreciate potential deleterious consequences of COVID-19 on the heart.

## Methods

In order to conduct this study, we recruited patients and controls from the general population of Columbia University Irving Medical Center (CUIMC)/New York-Presbyterian Hospital during the early months of 2021. Specifically, we recruited patients who had been seen at CUIMC who met the following inclusion criteria; convalescent COVID-19 patient (at least 4 weeks after symptoms and 2 weeks after hospital discharge if hospitalized), ability to give informed consent, willingness to undergo CMR and TTE, ability to perform 15 s breathe hold, and age 18 or older. These patients were not specifically planned to undergo cardiac testing and further cardiac imaging. Patients self-enrolled in the study after being contacted from a local database platform of subjects interested in clinical trial participation. We excluded patients who were pregnant, had chronic kidney disease stage 4 with estimated glomerular filtration ratio <30, incarcerated, known allergy to gadoterate, contraindication to CMR, known history of obstructive coronary artery disease with stenosis > 70% or fractional flow reserve <0.8, history of congestive heart failure prior to COVID-19, and severe valvular heart disease. We specifically aimed to enrol patients who did not have indications for cardiac magnetic resonance imaging (MRI) otherwise, in comparison to prior studies of unselected patients. For healthy controls, we ensure that they had no prior COVID, by asking to report prior COVID testing, medical records, and present proof of negative PCR/serology. The study protocol was approved by the institutional review board of our institution (IRB #AAAT0787). Informed consent was obtained from all patients.

We collected information on all subjects and patients including demographics, biometrics, presenting symptoms, any ongoing symptoms at time of testing, past medical history, and drew blood work for d-dimer, troponin, and haematocrit. We performed CMR in all recruited patients and TTE in all but one recruited patient due to the patient being lost to follow-up.

Transthoracic echocardiograms were performed using a commercially available system (iE 33, Philips, Andover, MA, USA) by an experienced cardiac sonographer or by a trained advanced imaging cardiology fellow blinded to the participant’s clinical information, following a standardized protocol. Tests were interpreted by a single experienced reader blinded to the subject’s clinical information. The dimensions of the cardiac chambers were measured according to the recommendations of the American Society of Echocardiography.^43^ Left ventricular ejection fraction (LVEF) was calculated using the modified biplane Simpson’s rule or visual estimation when appropriate. Left ventricular (LV) mass was calculated with a validated method and indexed for body surface area to generate LV mass index.^44^ LV diastolic function was assessed by combined spectral Doppler (*E* wave and *A* wave) and annular tissue Doppler (*E*′) evaluation; tissue Doppler S′ (systolic) annular velocities were measured as additional parameters of LV and right venticular (RV) systolic function. Echocardiographic variables of interest included indices of LV morphology, systolic and diastolic function; presence of valve disease; right ventricular dimension and function; left atrial and right atrial dimensions; presence of pericardial effusion.

We performed CMR using a 3T SIGNA™ Premier scanner (general electric [GE] HealthCare, Waukesha, WI, USA) with gadoterate meglumine (Clariscan, GE HealthCare) and using a 30-channel AIR Anterior coil and embedded Posterior coil (GE HealthCare). We performed functional assessment using a balanced Steady State Free Precession cine sequence for left ventricular and right ventricular (RV) function. Myocardial characterization was performed using T2-weighted imaging with double inversion recovery fast spin echo (FSE), T1 mapping using native modified Look Locker inversion (MOLLI-5(3)3) recovery sequence, gadolinium enhanced T1 mapping using MOLLI-4(1)3(1)2 and extracellular volume (ECV) fraction assessment, T2 mapping using a multi-echo FSE sequence, and late gadolinium enhancement (LGE) with phase-sensitive inversion recovery. CMR analysis was performed using cvi v5.11 (Circle Cardiovascular Imaging, Calgary). LGE was performed approximately 10 min after gadoterate injection. For extracellular volume measurements, post-contrast T1 mapping was performed >10 min after contrast injection based on Society for Cardiovascular Magnetic Resonance protocol recommendations.^45^

Each CMR study was read and processed independently by two trained expert cardiac radiologists with sub-specialization in cardiothoracic imaging and approximately 6 and 16 years each of experience independently reading studies. Parametric mapping was performed using regions of interest in the basal, mid-ventricular, and apical septa and averaged. ECV was calculated using the standard formula, specifically

$$\begin{array}{r} \mathrm{ECV}=(1-\mathrm{haematocrit})\frac{\frac{1}{T1\_\mathrm{myocardiu}m_{\mathrm{postcontrast}}}-\frac{1}{T1\_\mathrm{myocardiu}m_{\mathrm{precontrast}}}}{\frac{1}{T1\_\mathrm{bloo}d_{\mathrm{postcontrast}}}-\frac{1}{T1\_\mathrm{bloo}d_{\mathrm{precontrast}}}} \end{array}\;$$

CMR measurements included the following: left ventricular ejection fraction (LVEF), left ventricular end-diastolic volume index (LVEDVi), left ventricular mass index (LVMi), right ventricular ejection fraction (RVEF), right ventricular end diastolic volume index (RVEDVi), T1 mapping, T2 mapping, and extracellular volume fraction (ECV). In the final analysis, we averaged together quantitative variables except when the variables were more than 15% discrepant. When there were discrepant readings for qualitative and quantitative variables, we conducted an arbitration process in which a third independent reader with 16 years of experience met with the two original readers to decide upon a consensus read. In this way, final values for all variables across study population were determined.

Basic statistical analysis was carried out in R 1.4.1717. We performed univariate Student’s *t*-tests for continuous and *χ*^2^ tests for categorical variables. *P* < 0.05 was considered significant. Tests of normality were performed, and if data were distributed non-normally, the Kruskal–Wallis test was used for continuous variables. Rosner’s generalized extreme Studentized deviate test for outliers was performed with an alpha of 0.05 (allowable Type 1 error) and k of 3 (assumed number of outliers to assess for statistically significant outliers in the data set stratified by case and control). Violin plots were also generated for assessment of outliers and distribution, which show the data points within the outline of the distribution of the data displayed vertically on each axis.

## Results

We recruited a total of 52 participants for our study population, including 40 COVID-19 cases and 12 healthy controls (*Table 1*). Cases and controls were closely matched in age (median 46 vs. 40), sex (50% male), body mass index (median 27 vs. 26), and ethnicity (58% white, 25% Black) with 42% identifying as Hispanic ethnicity, without significant difference between these two subgroups. In general, these patients did not have substantial past medical history especially with regard to pre-existing cardiovascular disease. The presenting symptoms of the COVID patients were predominantly fever and dyspnoea although some also presented with chest pain. One patient self-identified themselves as having possible inflammation of the heart muscle, but this patient was not hospitalized for myocarditis, did not have imaging suggestive of this at the time of COVID, did not receive treatment for myocarditis, and has no proven diagnosis of myocarditis. Fourteen of the COVID-19 patients (35%) had illness requiring hospitalization, and seven (17.5%) of them had required intubation. At the time of presentation, a minority of patients had been experiencing ongoing chest pain, dyspnoea, and palpitations. No patient presented with acute coronary syndrome, acute aortic syndrome, venous thromboembolic phenomenon, or fulminant myocarditis. At time of study participation with imaging and blood work, a median of 308 days had passed from the onset of symptomatic COVID-19 (*Table 1*). At the time of scanning, no significant difference was detected in high sensitivity troponin T assay or d-dimer measurement between cases and controls with both being within normal limits for the standard lab ranges. At time of presentation for COVID, given that most patients were not specifically recruited from the hospital or for cardiac injury, specific biomarkers are not available in most of these patients (see Supplementary data online, *Table S1*). Furthermore, few patients received specific treatments for COVID such as remdesivir, steroids, and tocilizumab given the early time point in the pandemic prior to evidence of such therapies being widely available.

**Table 1:** Baseline characteristics of study participants

	COVID-19 negative (*N* = 12)	COVID-19 positive (*N* = 40)	*P*-value
Time from diagnosis to scan (days)	N/A	308 (225–391)	N/A
Baseline characteristics			
Age (years)	40 (27–55)	46 (34–65)	0.17
Female	6 (50%)	20 (50%)	1
Body mass index	26 (24–31)	27 (24–32)	0.59
White	7 (58%)	18 (45%)	0.32
Black	3 (25%)	11 (28%)	0.32
Hispanic	5 (42%)	19 (48%)	0.28
High-sensitivity Troponin-T	6.5 (5–14)	5.5 (5–11)	0.76
D-Dimer	0.15 (0–0.4)	0.37 (0–0.6)	0.07
Past medical history			
Atrial fibrillation	0 (0%)	0 (0%)	—
Chronic kidney disease	0 (0%)	0 (0%)	—
Chronic obstructive pulmonary disease	0 (0%)	0 (0%)	—
Coronary artery disease	0 (0%)	2 (5%)	—
Diabetes	0 (0%)	7 (18%)	0.28
Heart failure	0 (0%)	0 (0%)	—
Hypertension	0 (0%)	10 (25%)	0.13
Valvular disease	0 (0%)	0 (0%)	—
Prior myocardial infarction	0 (0%)	2 (5%)	1
COVID symptoms			
Fever	N/A	27 (68%)	N/A
Chest pain	N/A	11 (28%)	N/A
Dyspnoea	N/A	23 (57.5%)	N/A
Myocarditis	N/A	1 (2.5%)	N/A
Hospitalized	N/A	14 (35%)	N/A
Intubated	N/A	7 (17.5%)	N/A
Post-COVID symptoms			
Chest pain	N/A	2 (5%)	N/A
Dyspnoea	N/A	7 (17.5%)	N/A
Palpitations	N/A	4 (10%)	N/A

Values reported as median (interquartile range) or *N* (%).

N/A, not applicable.

On TTE measurements of cardiac function and structure, there were no significant differences in LVEF, left ventricular end-diastolic diameter, LV mass, or LV wall motion abnormalities between COVID-19 cases and controls (*Table 2*). There was no significant difference in left atrial diameter or markers of LV diastolic function such as the *E*/*E*′ ratio, and no significant difference in RV size, right atrial size, and RV function. However, slightly increased right ventricular systolic pressure was noted in COVID-19 patients compared to controls (median 26.5 vs. 22.5 mmHg, *P* = 0.03). No patient was found to have significant valvular disease. Echocardiographic findings stratified by non-hospitalized vs. hospitalized patients, as a marker of COVID-19 severity, are summarized in *Table 3*. While LVEF, LVEDd, LV mass, and segmental wall motion abnormalities were similar and normal between the groups, we did find a slightly increased thickness of interventricular septum thickness (IVS) (median 1.06 vs. 0.91 cm, *P* = 0.019) and larger left atrium (LA) diameter (median 3.26 vs. 3.83 mmHg, *P* = 0.002) between COVID-19 patients and controls. In terms of measures of diastolic dysfunction, as overall lower *E* (median 61 vs. 73, *P* = 0.007) and higher *A* (70 vs. 62, *P* = 0.03) was noted in COVID-19 survivors, though the *E*/*E*′ ratio was not significantly different and within normal limits for both groups (5.73 vs. 5.78, *P* = 0.47). Similar to the controls compared to the COVID-19 patients, RVSP was higher in the hospitalized COVID-19 group (30 vs. 25 mmHg, *P* = 0.01) with otherwise no significant differences in right atrial (RA) size, RV dysfunction, or valve disease.

**Table 2:** Echocardiographic characteristics of study participants

	COVID-19 negative (*N* = 12)	COVID-19 positive (*N* = 39)^a^	*P*-value
LVEF (%)	58 (57–60)	60 (54–63)	0.13
LVEDd (cm)	4.30 (4–4.5)	4.40 (4.1–4.7)	0.44
LVESd (cm)	3.14 (2.9–3.2)	3.00 (2.9–3.3)	0.87
IVS (cm)	0.98 (0.92–1.02)	0.99 (0.9–1.1)	0.70
PW (cm)	0.95 (0.87–1.03)	0.94 (0.86–1.00)	0.66
LV mass index (g/m^2^)	69.3 (61–80)	64.4 (61–88)	0.45
WMA	0 (0)	0 (0)	—
LV S′ (cm/s)	9.0 (9–11)	10.0 (9–12)	0.31
LA diameter (cm)	3.3 (3.2–3.8)	3.4 (3.2–3.9)	0.63
*E* (cm/s)	73 (68–77)	71 (61–78)	0.32
*A* (cm/s)	61 (50–76)	64 (51–71)	0.95
*E*/*A* ratio	1.34 (1.01–1.51)	1.14 (0.82–1.44)	0.34
*E*′ (cm/s)	11.5 (10–13)	12.0 (10–15)	0.92
*E*/*E*′ ratio	6.60 (6–7.2)	5.78 (5.3–7.4)	0.35
RV enlargement	4 (33)	4 (11)	0.15
RV S′ (cm/s)	11 (11–14)	13 (11–15)	0.17
RV dysfunction	1 (8)	0 (0)	0.54
RVSP (mmHg)	22.5 (22–23)	26.5 (25–29)	0.03
RA enlargement	3 (25)	1 (3)	0.08
Pericardial effusion	1 (8.3)	2 (5.1)	1

Values reported as median (interquartile range) or *N* (%).

LVEF, left ventricular ejection fraction; LVEDd, left ventricular end-diastolic diameter; LVESd, left ventricular end-systolic diameter; IVS, interventricular septum thickness; PW, posterior wall thickness; LV, left ventricle; WMA, wall motion abnormalities; LA, left atrium; RA, right atrial; RV, right ventricle; RVSP, right ventricular systolic pressure.

^a^TTE was not performed in one COVID-19 patient due to loss of follow-up.

**Table 3:** Echocardiographic characteristics of hospitalized vs. non-hospitalized COVID positive participants

	Non-hospitalized (*N* = 26)	Hospitalized (*N* = 13)	*P*-value
LVEF (%)	60 (58–62)	60 (57–63)	0.62
LVEDd (cm)	4.35 (4.1–4.7)	4.50 (4.2–4.6)	0.73
LVESd (cm)	2.98 (2.84–3.29)	3.19 (2.91–3.35)	0.52
IVS (cm)	0.91 (0.84–1.06)	1.06 (1.00–1.11)	0.02
PW (cm)	0.90 (0.84–0.98)	0.99 (0.91–1.10)	0.04
LV mass index (g/m^2^)	68.7 (62–82)	69.9 (62–92)	0.79
WMA	0 (0)	0 (0)	—
LV S′ (cm/s)	10.0 (9–12)	10.0 (8.5–11.5)	0.33
LA diameter (cm)	3.26 (3.00–3.56)	3.83 (3.77–4.06)	0.002
*E* (cm/s)	73 (69–80)	61 (55–70)	0.007
*A* (cm/s)	62 (51–66)	70 (66–77)	0.03
*E*/*A* ratio	1.29 (1.06–1.52)	0.81 (0.78–1.02)	0.002
*E*′ (cm/s)	12.5 (10–15)	11.0 (9.5–13.5)	0.40
*E*/*E*′ ratio	5.78 (5.4–8.1)	5.73 (5–6.3)	0.47
RV enlargement	2 (8)	2 (15)	0.88
RV S′ (cm/s)	14 (13–15)	11 (10–13)	0.07
RV dysfunction	0 (0)	0 (0)	—
RVSP (mmHg)	25.5 (24–27)	30.0 (29.5–30.3)	0.011
RA enlargement	1 (4)	0 (0)	1
Pericardial effusion	2 (7.7)	0 (0)	0.79

Values reported as median (interquartile range) or *N* (%).

N/A, not applicable; LVEF, left ventricular ejection fraction; LVEDd, left ventricular end diastolic diameter; IVS, interventricular septum thickness; LV, left ventricle; WMA, wall motion abnormality; LA, left atrium; RA, right atrial; RV, right ventricle; RVSP, right ventricular systolic pressure.

**Figure 1:**
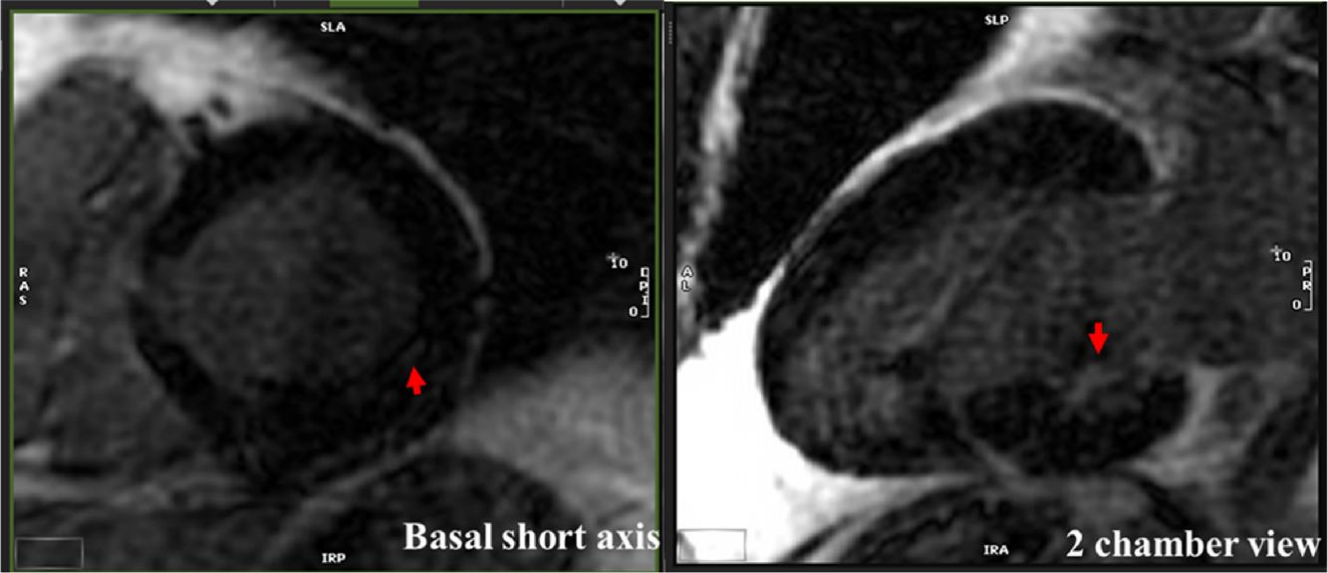
Late gadolinium in cardiac magnetic resonance imaging scan of COVID-19 patient. Late gadolinium in the basal inferior wall in the only patient in the cohort with enhancement at 10 months post-hospitalization and intubation for COVID-19 This scan was performed using a 3T GE SIGNA™ Premier scanner with late gadolinium enhancement with phase-sensitive inversion recovery, obtained 10 min after administration of gadoterate meglumine (Clariscan, GE Healthcare). Arrow points to LGE.

**Figure 2:**
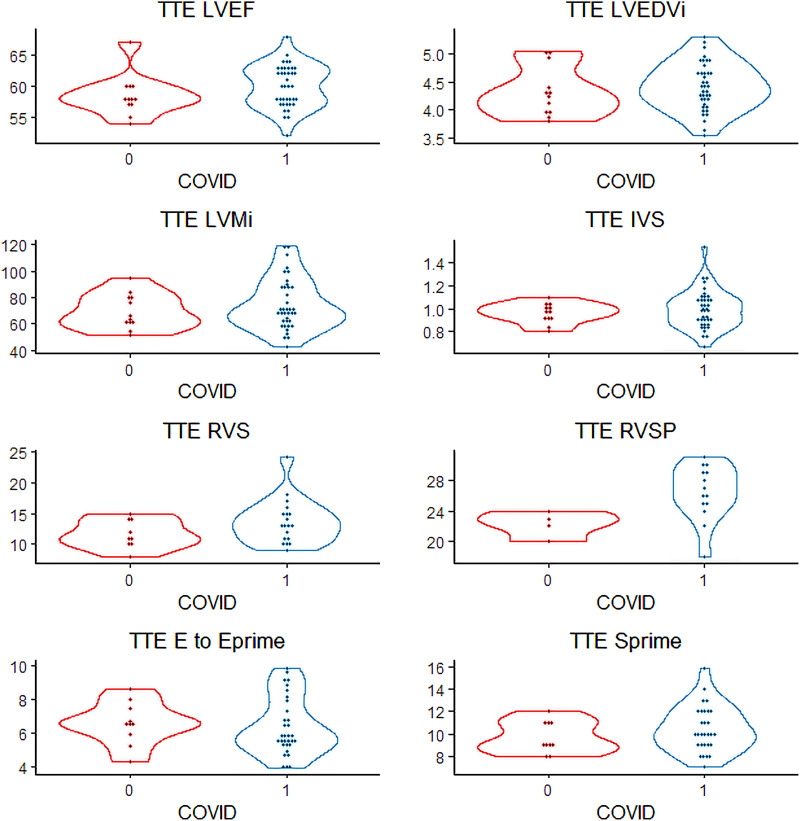
Violin plots of transthoracic echocardiogram characteristics of COVID-19 patients vs. controls. Distribution of key echocardiographic characteristics between cases (1, blue) and controls (2, red) including LVEF, left ventricular ejection fraction; LVEDVi, left ventricular end diastolic volume index; LVMi, left ventricular mass index; IVS, interventricular septum thickness; RVS, right ventricular S; RVSP, right ventricular systolic pressure; *E* to *E*′, *E* to Eprime; S′, Sprime. These violin plots show data points within the outline of the distribution of the data plotted vertically.

**Figure 3:**
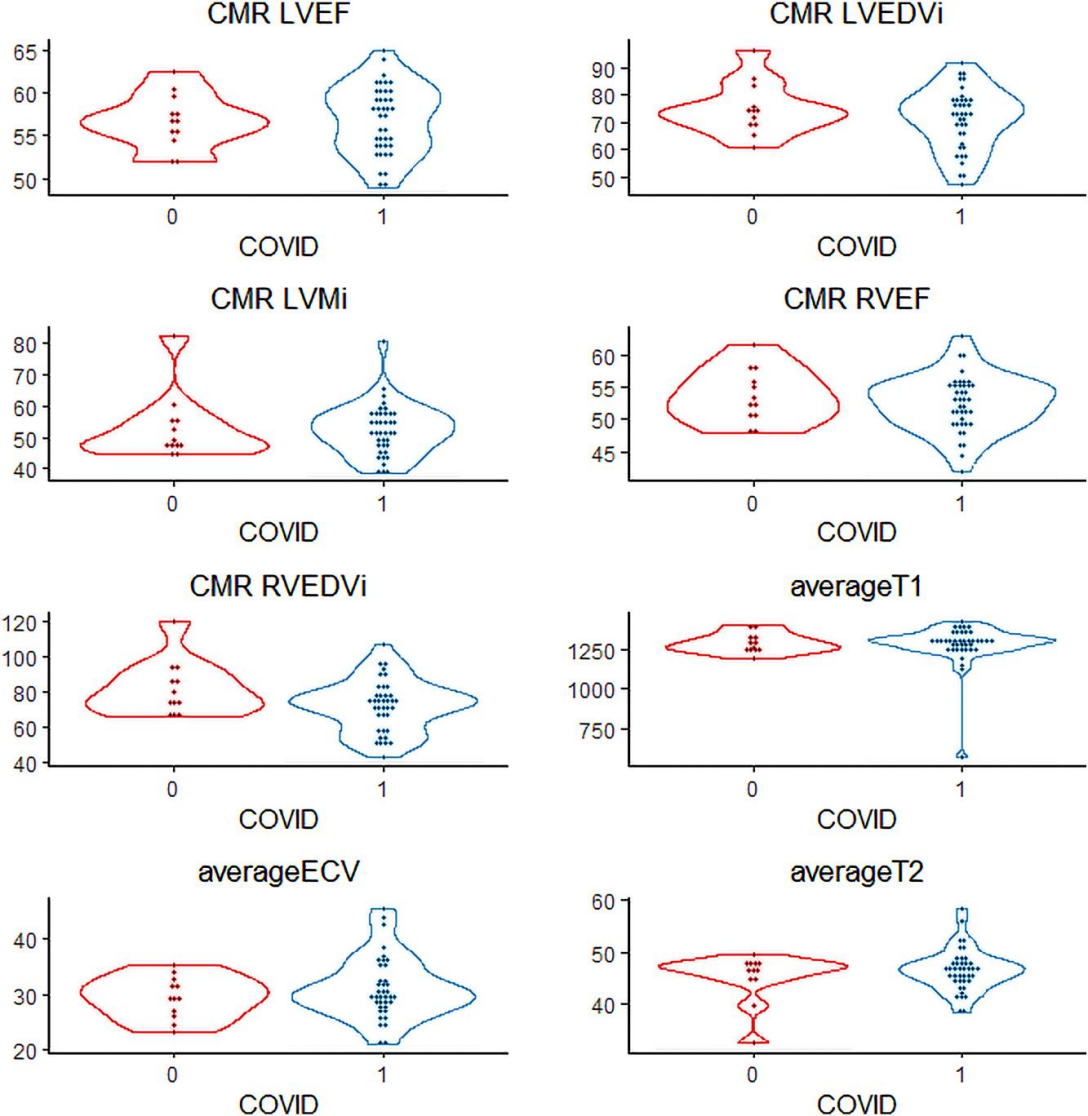
Violin plots of cardiac magnetic resonance characteristics of COVID-19 patients vs. controls. Distribution of key CMR characteristics between cases (1, blue) and controls (2, red) including LVEF (left ventricular ejection fraction), LVEDVi (left ventricular end diastolic volume index), LVMi (left ventricular mass index), RVEF (right ventricular ejection fraction), RVEDVi (right ventricular end diastolic volume index), T1, T2, and extracellular volume fraction.

CMR was performed not only at myocardial macrostructure and function but also extract measures of tissue microstructure. In comparing COVID-19 patients to controls (*Table 4*), we found overall no significant differences; in particular LVEF, left ventricular end-diastolic index, right ventricular ejection fraction, and right ventricular end-diastolic volume index were similar and no wall motion abnormalities were noted. One patient in the COVID-19 positive group was noted to have a mild pericardial effusion. On assessment of tissue microstructure, there was no significant difference in median T1 (1278 vs. 1306 ms), T2 (47 vs. 47 ms), and ECV fraction (29 vs. 30) between COVID cases and controls. In only one case was LGE observed, which involved only 1 of 17 segments, specifically the mid-myocardial basal inferior wall (*Figure 1*). This patient initially presented with fever and dyspnoea, was hospitalized, and intubated for respiratory failure, with no initial acute cardiac concerns identified, though he remained symptomatic with shortness of breath at the time of the study, albeit without requirement for long-term oxygen therapy. There was no significant difference in these parameters between hospitalized and non-hospitalized cases (*Table 5*). We generated distribution plots of key TTE and CMR parameters (*Figures 2* and *3*) and formal statistical testing with Rosner’s tests for outliers (*Table 6*). We found few outliers in both cases and controls overall.

**Table 4:** CMR characteristics of study participants

	COVID-19 negative (*N* = 12)	COVID-19 positive (*N* = 40)	*P*-value
LVEF (%)	57 (55–58)	58 (54–60)	0.78
LVEDV index	74 (70–78)	74 (63–78)	0.28
LV mass index	49 (51–57)	52 (48–57)	0.77
Wall motion abnormality	0 (0)	0 (0)	—
RVEF (%)	53 (51–57)	53 (50–55)	0.35
RVEDV index	78 (72–88)	74 (61–78)	0.12
Pericardial effusion	0 (0)	1 (3)	1
T1 (ms)	1278 (1250–1325)	1306 (1254–1327)	0.56
T2 (ms)	47 (45–48)	47 (45–48)	0.90
ECV	29 (27–31)	30 (28–34)	0.44
LGE	0 (0)	1 (3)	1

Values reported as median (interquartile range) or *N* (%).

N/A, not applicable; LVEF, left ventricular ejection fraction; LVEDV, left ventricular end-diastolic volume; RVEF, right ventricular ejection fraction; RVEDV, right ventricular end-diastolic volume; ECV, extracellular volume fraction; LGE, late gadolinium enhancement.

**Table 5:** CMR characteristics of hospitalized vs. non-hospitalized COVID + participants

	Non-hospitalized (*N* = 26)	Hospitalized (*N* = 14)	*P*-value
LVEF (%)	59 (55–60)	54 (53–58)	0.12
LVEDV index	74 (65–78)	70 (60–75)	0.29
LV mass index	54.7 (47–58)	51.0 (50–54)	0.11
Wall motion abnormality	0 (0)	0 (0)	—
RVEF (%)	54.3 (51–55)	51 (49–53)	0.11
RVEDV index	75.5 (67–82)	71.5 (58–76)	0.20
Pericardial effusion	1 (4)	0 (0)	1
T1 (ms)	1306 (1250–1319)	1305 (1267–1351)	0.41
T2 (ms)	46.7 (45–49)	45.8 (42–48)	0.23
ECV	30.4 (29–36)	28.2 (27–31)	0.08
LGE	0 (0)	1 (11)	0.59

Values reported as median (interquartile range) or *N* (%).

N/A, not applicable; LVEF, left ventricular ejection fraction; LVEDV, left ventricular end diastolic volume; WMA, wall motion abnormality; RVEF, right ventricular ejection fraction; RVEDV, right ventricular end diastolic volume; ECV, extracellular volume fraction; LGE, late gadolinium enhancement.

**Table 6:** Statistical outliers using Rosner’s test

Modality	Variable	COVID	Value	Average value
TTE	IVS (cm)	Case	1.54	0.99
TTE	LVEF (%)	Control	67	58
TTE	*E* (cm/s)	Control	111	73
CMR	LVMi	Case	80.6	52
CMR	T1 (ms)	Case	572	1306
CMR	LVMi	Control	82.3	49
CMR	RVEDVi	Control	119	78
CMR	T2 (ms)	Control	32.6	47
CMR	T2 (ms)	Control	39.9	47

Variables shown here include: including left ventricular ejection fraction (LVEF), left ventricular mass index (LVMi), interventricular septum thickness (IVS), right ventricular end diastolic volume index (RVEDVi), E, T1, and T2.

TTE, transthoracic echocardiography; CMR, cardiac magnetic resonance.

## Discussion

In this cross-sectional study of a multi-ethnic cohort of patients recruited from the initial phase of the COVID-19 pandemic, comprehensive analysis of cardiac structure and function using multimodality imaging with transthoracic echocardiography and multi-sequence cardiac MRI reassuringly found no signs of clinically significant changes to the myocardium. Imaging was performed at least 6 months and on average 10 months after initial COVID-19 infection during the initial phase of the pandemic in patients who were not otherwise scheduled for TTE or CMR, allowing an examination of patients at a distinctly remote time point from initial presentation, one of the longest term studies in the literature at this time. Moreover, this study is unique in its inclusion of a multi-ethnic cohort, with a majority of participants being Hispanic and/or Black.

Our findings did suggest a slight but significant increase in pulmonary hypertension in COVID-19 patients, particularly those who were sicker. LA diameter and IVS thickness were also different, but it is unclear that these are clinically relevant. However, for these patients, it does not seem that these differences significantly impact RV and LV functions at this time interval of 10 months after initial illness.

In some ways, these findings are not expected given the previous literature, though findings have been varied. An important early study in the midst of the early pandemic by Puntmann *et al.*^5^ performed CMR prospectively in 100 patients including 33% hospitalized about 71 days after initial COVID infection. In this study of German patients, it was found that 78% of patients had ongoing myocardial inflammation independent of pre-existing condition, severity, and overall course of acute illness. Such findings in this and other studies^8,25,30,34^ at early time points after COVID-19 of weeks to months have raised concerns that ongoing myocardial inflammation and damage may have serious long-term complications for cardiovascular health of many COVID-19 patients. Such alarming early data motivated the need for ongoing longer term follow-up studies, such as the present study.

CMR has been used in just a few studies to characterize long-term myocardial consequences of COVID-19. Cassar *et al.*^16^ used CMR in combination with cardiopulmonary exercise testing to study COVID-19 patients who were admitted and had ongoing cardiopulmonary symptoms about 3–6 months after initial infection; in their cohort, they found little evidence of persistent myocardial abnormalities that correlated with patient symptoms. Hanneman *et al.*^24^ studied prospectively mostly mild COVID-19 patients about 2–4 months after initial infection using PET and CMR; in this cohort, they found that about 17% of patients had focal FDG-PET uptake that correlated with T1, T2, LGE on CMR with mildly worsened contractile function, and strain initially at 2 months which had subsequently resolved within 4 months at follow-up. Joy *et al.* performed a 6-month prospective case–control studies of health care workers who had predominantly asymptomatic and mild COVID, finding no abnormal markers of COVID infection on CMR.^22^ Li *et al.*^40^ published a prospective observational cohort at 5–6 months post-infection finding only one patient with LGE among 40 but identifying mildly elevated ECV and reduced global longitudinal strain in patients with moderate/severe COVID but not ongoing cardiac symptoms, demonstrating a subclinical manifestation of COVID-19. Tanacli *et al.*^46^ published a retrospective series of 32 patients with persistent cardiac symptoms about 5 months after initial COVID infection along with endomyocardial biopsy demonstrating that about 30% of these patients showed myocardial injury but only 9% meeting full Lake Louise criteria without any demonstrating true myocarditis on biopsy which was pursued in these patients.

We add to this literature by extending the time frame to over 10 months and including a multi-ethnic group of patients as seen here. Despite analysis of average tendencies and outliers, we do not detect specific differences in cases and controls at this time point. Overall, these data are reassuring that COVID-19 in a general population does not lead to significant cardiac damage and ongoing inflammation at this long-term follow-up. Significant differences may not have been found in our current study due to the longer time of follow-up after COVID infection and the focus on patients without presentation of acute cardiac illness. Given prior studies finding a high burden of findings in many patients during and soon after illness, it may be that there was transient myocardial involvement that had resolved at the time of this study, though longitudinal studies would be required to establish such findings.

We acknowledge limitations of this current study including a moderate sized cohort that may not be powered to detect subtle differences. However, it may be argued that subtle differences may not necessarily be of clinical significance. We also performed outlier analysis that would allow us to detect clinically significant deviations from the average tendency and found no significant outliers for COVID phenotypes. We also acknowledge that none of these patients presented with acute coronary syndrome, myocarditis, venous thromboembolism which have been reported as particularly serious cardiovascular complications that have been reported in acute COVID-19 infection and therefore represent a select group of patients with COVID-19. Our study population represented altogether a relatively young group of COVID-19 patients few comorbidities. While additional work should be done to investigate long-term cardiac outcomes in COVID-19 patients presenting with myocarditis, acute coronary syndromes and thromboembolism as well as those with post-acute sequelae of COVID-19, it is reassuring that in this cohort of patients from the early virulent phase of the pandemic when there were no vaccinations there is limited lasting damage to the heart from COVID-19.

## Supplementary Material

qyad034_Supplementary_Data

## Data Availability

Data used in this study will not be publicly available.

## Lead author biography

Andrew Einstein is a multimodality cardiac imager and clinical cardiologist at Columbia University Irving Medical Center and NewYork-Presbyterian Hospital, where he a is a tenured professor of medicine, Director of Nuclear Cardiology, Cardiac computed tomography, and Cardiac MRI, and Director of the Advanced Cardiac Imaging Fellowship.

## References

[1] Altay S. COVID-19 myocarditis cardiac magnetic resonance findings in symptomatic patients. Acta Radiol 2022;63:1475–80. 34623175 10.1177/02841851211046502 PMC9548510

[2] Lala A, Johnson KW, Januzzi JL, Russak AJ, Paranjpe I, Richter F et al Prevalence and impact of myocardial injury in patients hospitalized with COVID-19 infection. J Am Coll Cardiol 2020;4:533–46. 10.1016/j.jacc.2020.06.007 PMC7279721 32517963

[3] Montero-Cabezas JM, Córdoba-Soriano JG, Díez-Delhoyo F, Abellán-Huerta J, Girgis H, Rama-Merchán JC et al Angiographic and clinical profile of patients with COVID-19 referred for coronary angiography during SARS-CoV-2 outbreak: results from a collaborative, European, multicenter registry. Angiology 2022;73:112–9. 34318686 10.1177/00033197211028760

[4] Faridi KF, Hennessey KC, Shah N, Soufer A, Wang Y, Sugeng L et al Left ventricular systolic function and inpatient mortality in patients hospitalized with coronavirus disease 2019 (COVID-19). J Am Soc Echocardiogr 2020;33:1414–5. 32951969 10.1016/j.echo.2020.08.016 PMC7442910

[5] Puntmann VO, Carerj ML, Wieters I, Fahim M, Arendt C, Hoffmann J et al Outcomes of cardiovascular magnetic resonance imaging in patients recently recovered from coronavirus disease 2019 (COVID-19). JAMA Cardiol 2020;5:1265–73. 32730619 10.1001/jamacardio.2020.3557 PMC7385689

[6] Brito D, Meester S, Yanamala N, Patel HB, Balcik BJ, Casaclang-Verzosa G et al High prevalence of pericardial involvement in college student athletes recovering from COVID-19. JACC Cardiovasc Imaging 2021;14:541–55. 33223496 10.1016/j.jcmg.2020.10.023 PMC7641597

[7] Wang H, Li R, Zhou Z, Jiang H, Yan Z, Tao X et al Cardiac involvement in COVID-19 patients: mid-term follow up by cardiovascular magnetic resonance. J Cardiovasc Magn Reson 2021;23:14. 33627143 10.1186/s12968-021-00710-x PMC7904320

[8] Wojtowicz D, Dorniak K, Ławrynowicz M, Rejszel-Baranowska J, Fijałkowska J, Kulawiak-Gałąska D et al Spectrum of lesions visualized in cardiac magnetic resonance imaging in COVID-19-related myocarditis: findings from a pilot study of the TRICITY-CMR trial. Cardiol J 2021;28:976–8. 34708861 10.5603/CJ.a2021.0139 PMC8747819

[9] Singh T, Kite TA, Joshi SS, Spath NB, Kershaw L, Baker A et al MRI and CT coronary angiography in survivors of COVID-19. Heart 2022;108:46–53. 34615668 10.1136/heartjnl-2021-319926 PMC8503921

[10] Dweck MR, Bularga A, Hahn RT, Bing R, Ken Lee K, Chapman AR et al Global evaluation of echocardiography in patients with COVID-19. Eur Heart J Cardiovasc Imaging 2020;21:949–58. 32556199 10.1093/ehjci/jeaa178 PMC7337658

[11] Stefanini GG, Montorfano M, Trabattoni D, Andreini D, Ferrante G, Ancona M et al ST-elevation myocardial infarction in patients with COVID-19: clinical and angiographic outcomes. Circulation 2020;141:2113–6. 32352306 10.1161/CIRCULATIONAHA.120.047525 PMC7302062

[12] Szekely Y, Lichter Y, Taieb P, Banai A, Hochstadt A, Merdler I et al Spectrum of cardiac manifestations in COVID-19: a systematic echocardiographic study. Circulation 2020;142:342–53. 32469253 10.1161/CIRCULATIONAHA.120.047971 PMC7382541

[13] Kawel-Boehm N, Hetzel SJ, Ambale-Venkatesh B, Captur G, Francois CJ, Jerosch-Herold M et al Reference ranges (“normal values”) for cardiovascular magnetic resonance (CMR) in adults and children: 2020 update. J Cardiovasc Magn Reson 2020;22:87. 33308262 10.1186/s12968-020-00683-3 PMC7734766

[14] Salerno M, Kwong RY. CMR in the era of COVID-19: evaluation of myocarditis in the subacute phase. JACC Cardiovasc Imaging 2020;13:2340–2. 32771570 10.1016/j.jcmg.2020.06.013 PMC7332904

[15] Ferreira VM, Schulz-Menger J, Holmvang G, Kramer CM, Carbone I, Sechtem U et al Cardiovascular magnetic resonance in nonischemic myocardial inflammation: expert recommendations. J Am Coll Cardiol 2018;72:3158–76. 30545455 10.1016/j.jacc.2018.09.072

[16] Cassar MP, Tunnicliffe EM, Petousi N, Lewandowski AJ, Xie C, Mahmod M et al Symptom persistence despite improvement in cardiopulmonary health—insights from longitudinal CMR, CPET and lung function testing post-COVID-19. EClinicalMedicine 2021;41:101159. 34693230 10.1016/j.eclinm.2021.101159 PMC8527025

[17] Clark DE, Parikh A, Dendy JM, Diamond AB, George-Durrett K, Fish FA et al COVID-19 myocardial pathology evaluation in athletes with cardiac magnetic resonance (COMPETE CMR). Circulation 2021;143:609–12. 33332151 10.1161/CIRCULATIONAHA.120.052573 PMC7864610

[18] Viskin D, Topilsky Y, Aviram G, Mann T, Sadon S, Hadad Y et al Myocarditis associated with COVID-19 vaccination: echocardiography, cardiac tomography, and magnetic resonance imaging findings. Circ Cardiovasc Imaging 2021;14:e013236. 34428917 10.1161/CIRCIMAGING.121.013236 PMC8478100

[19] Clark DE, Dendy JM, Li DL, Crum K, Dixon D, George-Durrett K et al Cardiovascular magnetic resonance evaluation of soldiers after recovery from symptomatic SARS-CoV-2 infection: a case–control study of cardiovascular post-acute sequelae of SARS-CoV-2 infection (CV PASC). J Cardiovasc Magn Reson 2021;23:106. 34620179 10.1186/s12968-021-00798-1 PMC8495668

[20] Martinez MW, Tucker AM, Bloom OJ, Green G, Difiori JP, Solomon G et al Prevalence of inflammatory heart disease among professional athletes with prior COVID-19 infection who received systematic return-to-play cardiac screening. JAMA Cardiol 2021;6:745–52. 33662103 10.1001/jamacardio.2021.0565 PMC7934073

[21] Ponsiglione A, Nappi C, Imbriaco M, Ascione R, Megna R, Petretta M et al Cardiac magnetic resonance imaging during the COVID-19 pandemic: a southern Italian single-center experience. Eur J Radiol Open 2021;8:100319. 33392363 10.1016/j.ejro.2020.100319 PMC7764388

[22] Joy G, Artico J, Kurdi H, Seraphim A, Lau C, Thornton GD et al Prospective case-control study of cardiovascular abnormalities 6 months following mild COVID-19 in healthcare workers. JACC Cardiovasc Imaging 2021;14:2155–66. 33975819 10.1016/j.jcmg.2021.04.011 PMC8105493

[23] Hatipoglu S, Lyon AR, Pennell DJ. CMR unveiling the cause of post COVID-19 infection chest pain. Int J Cardiovasc Imaging 2021;37:2025–6. 33507427 10.1007/s10554-021-02161-y PMC7841042

[24] Hanneman K, Houbois C, Schoffel A, Gustafson D, Iwanochko RM, Wintersperger BJ et al Combined cardiac fluorodeoxyglucose-positron emission tomography/magnetic resonance imaging assessment of myocardial injury in patients who recently recovered from COVID-19. JAMA Cardiol 2022;7:298–308. 35019953 10.1001/jamacardio.2021.5505 PMC8756363

[25] Moulson N, Petek BJ, Drezner JA, Harmon KG, Kliethermes SA, Patel MR et al SARS-CoV-2 cardiac involvement in young competitive athletes. Circulation 2021;144:256–66. 33866822 10.1161/CIRCULATIONAHA.121.054824 PMC8300154

[26] Ojha V, Verma M, Pandey NN, Mani A, Malhi AS, Kumar S et al Cardiac magnetic resonance imaging in coronavirus disease 2019 (COVID-19): a systematic review of cardiac magnetic resonance imaging findings in 199 patients. J Thorac Imaging 2021;36:73–83. 33306666 10.1097/RTI.0000000000000574

[27] Seidel F, Kuehne T, Kelle S, Doeblin P, Zieschang V, Tschoepe C et al Cardiovascular magnetic resonance findings in non-hospitalized paediatric patients after recovery from COVID-19. ESC Hear Fail 2021;8:5583–8. 10.1002/ehf2.13678 PMC8652950 34704672

[28] Barris DM, Keelan J, Ahluwalia N, Jhaveri S, Cohen J, Stern K et al Midterm outcomes and cardiac magnetic resonance imaging following multisystem inflammatory syndrome in children. J Pediatr 2022;241:237–241.e1. 34687695 10.1016/j.jpeds.2021.10.009

[29] Bartoszek M, Małek ŁA, Barczuk-Falęcka M, Brzewski M. Cardiac magnetic resonance follow-up of children after pediatric inflammatory multisystem syndrome temporally associated with SARS-CoV-2 with initial cardiac involvement. J Magn Reson Imaging 2022;55:883–91. 34327751 10.1002/jmri.27870 PMC8426796

[30] Rajpal S, Tong MS, Borchers J, Zareba KM, Obarski TP, Simonetti OP et al Cardiovascular magnetic resonance findings in competitive athletes recovering from COVID-19 infection. JAMA Cardiol 2021;6:116–8. 32915194 10.1001/jamacardio.2020.4916 PMC7489396

[31] Galea N, Marchitelli L, Pambianchi G, Catapano F, Cundari G, Birtolo LI et al T2-mapping increase is the prevalent imaging biomarker of myocardial involvement in active COVID-19: a cardiovascular magnetic resonance study. J Cardiovasc Magn Reson 2021;23:68. 34107985 10.1186/s12968-021-00764-x PMC8189727

[32] Huang L, Zhao P, Tang D, Zhu T, Han R, Zhan C et al Cardiac involvement in patients recovered from COVID-2019 identified using magnetic resonance imaging. JACC Cardiovasc Imaging 2020;13:2330–9. 32763118 10.1016/j.jcmg.2020.05.004 PMC7214335

[33] Kelle S, Bucciarelli-Ducci C, Judd RM, Kwong RY, Simonetti O, Plein S et al Society for Cardiovascular Magnetic Resonance (SCMR) recommended CMR protocols for scanning patients with active or convalescent phase COVID-19 infection. J Cardiovasc Magn Reason 2020;22:61. 10.1186/s12968-020-00656-6 PMC7467754 32878639

[34] Daniels CJ, Rajpal S, Greenshields JT, Rosenthal GL, Chung EH, Terrin M et al Prevalence of clinical and subclinical myocarditis in competitive athletes with recent SARS-CoV-2 infection: results from the big ten COVID-19 cardiac registry. JAMA Cardiol 2021;6:1078–87. 34042947 10.1001/jamacardio.2021.2065 PMC8160916

[35] Kotecha T, Knight DS, Razvi Y, Kumar K, Vimalesvaran K, Thornton G et al Patterns of myocardial injury in recovered troponin-positive COVID-19 patients assessed by cardiovascular magnetic resonance. Eur Heart J 2021;42:1866–78. 33596594 10.1093/eurheartj/ehab075 PMC7928984

[36] Starekova J, Bluemke DA, Bradham WS, Eckhardt LL, Grist TM, Kusmirek JE et al Evaluation for myocarditis in competitive student athletes recovering from coronavirus disease 2019 with cardiac magnetic resonance imaging. JAMA Cardiol 2021;6:945–50. 33443537 10.1001/jamacardio.2020.7444 PMC7809616

[37] Raafs AG, Ghossein MA, Brandt Y, Henkens MTHM, Kooi ME, Vernooy K et al Cardiovascular outcome 6 months after severe coronavirus disease 2019 infection. J Hypertens 2022;40:1278–87. 35221322 10.1097/HJH.0000000000003110

[38] Xie Y, Xu E, Bowe B, Al-Aly Z. Long-term cardiovascular outcomes of COVID-19. Nat Med 2022;28:583–90. 35132265 10.1038/s41591-022-01689-3 PMC8938267

[39] Petersen SE, Friedrich MG, Leiner T, Elias MD, Ferreira VM, Fenski M et al Cardiovascular magnetic resonance for patients with COVID-19. JACC Cardiovasc Imaging 2022;15:685–99. 34656482 10.1016/j.jcmg.2021.08.021 PMC8514168

[40] Li X, Wang H, Zhao R, Wang T, Zhu Y, Qian Y et al Elevated extracellular volume fraction and reduced global longitudinal strains in participants recovered from COVID-19 without clinical cardiac findings. Radiology 2021;299:E230–40. 33434112 10.1148/radiol.2021203998 PMC7808090

[41] Esposito A, Palmisano A, Natale L, Ligabue G, Peretto G, Lovato L et al Cardiac magnetic resonance characterization of myocarditis-like acute cardiac syndrome in COVID-19. JACC Cardiovasc Imaging 2020;13:2462–5. 32654966 10.1016/j.jcmg.2020.06.003 PMC7314439

[42] van Driest FY, Fejzovic V, Scholte AJHA, Jukema JW, Lamb HJ. COVID-19 associated perimyocarditis. Magn Reson Imaging 2021;84:132–4. 34626774 10.1016/j.mri.2021.08.012 PMC8492890

[43] Lang RM, Badano LP, Victor MA, Afilalo J, Armstrong A, Ernande L et al Recommendations for cardiac chamber quantification by echocardiography in adults: an update from the American Society of Echocardiography and the European Association of Cardiovascular Imaging. J Am Soc Echocardiogr 2015;28:1–39.e14. 25559473 10.1016/j.echo.2014.10.003

[44] Devereux RB, Reichek N. Echocardiographic determination of left ventricular mass in man. Anatomic validation of the method. Circulation 1977;55:613–8. 138494 10.1161/01.cir.55.4.613

[45] Kramer CM, Barkhausen J, Bucciarelli-Ducci C, Flamm SD, Kim RJ, Nagel E. Standardized cardiovascular magnetic resonance imaging (CMR) protocols: 2020 update. J Cardiovasc Magn Reson 2020;22:17. 32089132 10.1186/s12968-020-00607-1 PMC7038611

[46] Tanacli R, Doeblin P, Götze C, Zieschang V, Faragli A, Stehning C et al COVID-19 vs. Classical myocarditis associated myocardial injury evaluated by cardiac magnetic resonance and endomyocardial biopsy. Front Cardiovasc Med 2021;8:737257. 35004872 10.3389/fcvm.2021.737257 PMC8739473

