# Supplementary material for: Absence of long-term structural and functional cardiac abnormalities on multimodality imaging in a multi-ethnic group of COVID-19 survivors from the early stage of the pandemic
Source: Eur Heart J Imaging Methods Pract. 2023 Oct 26;1(2):qyad034. doi: 10.1093/ehjimp/qyad034 (PMC11195772; doi:10.1093/ehjimp/qyad034)
Supplement: qyad034_Supplementary_Data [file qyad034_Supplementary_Data.docx]

**Supplemental Table 1. Additional Characteristics of Patients at Time of COVID diagnosis (if available)**

|  | N ED/ Admitted | N ED/Admitted with +value | N hospitalized  with +value | N intubated  with +value |
| --- | --- | --- | --- | --- |
| HsTnT at COVID diagnosis | 12 | 4 | 4 | 1 |
| + = HsTnT > 12 | | | | |
|  |  |  |  |  |
| D-dimer at COVID diagnosis | 11 | 8 | 8 | 4 |
| + = d-dimer>0.4 |  |  |  |  |
|  |  |  |  |  |
| NTproBNP at COVID diagnosis | 9 | 7 | 7 | 4 |
| + = NtproBNP>450 | | | | |
|  |  |  |  |  |
| Patient receiving remdesivir | 7 | 7 | 7 | 1 |
| Patient receiving steroids | 8 | 8 | 8 | 2 |
| Patient receiving tocilizumab | 2 | 2 | 2 | 2 |

HsTnT denotes high sensitivity troponin t assay, which has a cutoff of 12 in the laboratory used.

D-dimer has a cutoff of 0.4 in the laboratory used.

NTproBNP has

Steroids include dexamethasone, prednisone, methylprednisolone.
